# Variation in type IV pilus stability modulates DNA uptake and biofilm formation

**DOI:** 10.1016/j.jbc.2025.110787

**Published:** 2025-10-06

**Authors:** Yafan Yu, Rabab Mahdi, Ahmad Al-Hilfy Leon, Nam Vo, Reese Lofgren, Jean Luc Mutabazi, Kurt H. Piepenbrink

**Affiliations:** 1 Department of Biochemistry, University of Nebraska-Lincoln, Lincoln, Nebraska, USA; 2 Department of Microbiology, University of Georgia, Athens, Georgia, USA; 3 Department of Food Science and Technology, University of Nebraska-Lincoln, Lincoln, Nebraska, USA; 4 Department of Chemistry, University of Nebraska-Lincoln, Lincoln, Nebraska, USA; 5 Nebraska Food for Health Center, University of Nebraska-Lincoln, Lincoln, Nebraska, USA; 6 Center for Integrated Biomolecular Communication, University of Nebraska-Lincoln, Lincoln, Nebraska, USA

**Keywords:** *Acinetobacter baumannii*, twitching motility, biofilm formation, DNA uptake, natural competence, type IV pili, molecular evolution, phylogenetic incongruence

## Abstract

Type IV pili (T4P) are helical filaments composed of protein subunits, which are produced by numerous taxa of bacteria, including *Acinetobacter*. T4P are extended out from the cell by extension of enzyme complexes, which extract subunits from the membrane and insert them into the base of the filament, but can also be retracted by reverse rotation catalyzed by a retraction enzyme. T4P have diverse functions, including twitching motility and DNA uptake, which require retraction, and host adhesion and bacterial aggregation, which do not require retraction. *Acinetobacter* bacteria, including International Clone I (IC-I) and International Clone II (IC-II) strains, show variable phenotypes in assays of T4P-dependent functions. Here, we show that this variation is the result of differentiation of T4P subtypes in *Acinetobacter*, which we defined based on the sequence of the major subunit, PilA. These subtypes show variable efficiency in pilus retraction between pilus subtypes, and from that, a differential balance between retraction-dependent and retraction-independent functions. In both naturally occurring *pilA* variants from the IC-I and IC-II groups and isogenic strains complemented with IC-I or IC-II *pilA*, the IC-I pilus subtype promotes greater twitching motility and DNA uptake, whereas the IC-II pilus subtype promotes biofilm formation while showing reduced capacity for DNA uptake and twitching motility, similar to a retraction-deficient mutant and consistent with the hypothesis that pilus retraction of the IC-II pilus is naturally deficient. This defect in retraction was sufficient to increase the level of piliation on the cell surface when we compared the yields of T4P sheared from the cell surface from IC-I *pilA* and IC-II *pilA* complements in an isogenic background. Complementation with IC-II *pilA* results in greater levels of surface PilA per cell than equivalent complementation with an IC-I *pilA* gene. In addition, direct comparisons of pilus stability between T4P isolated from IC-I *pilA* and IC-II *pilA* complements show greater thermostability for the IC-II pili, supporting the hypothesis that pilus stability can impede retraction and increase piliation.

Type IV filaments, encompassing type IV pili (T4P) and related fibers, Tad/Flp pili, and competence (com) pili, are found in a wide variety of bacterial taxa and show significant variation in both structure and function (1, 2, 3, 4, 5). However all these fibers are united by similarities in structure and function; they are assembled and extended by polymerizing protein subunits into a helical, extracellular assembly; they are adherent to DNA or other biomolecules; and they all appear to be retractile; that is, they can be depolymerized with the subunits returning to the membrane, shortening the fiber and potentially reeling in attached biomolecular cargo.

The divisions between com pili, T4P, and Tad/Flp pili are based on structural differences between the subunits (4), but clear functional differences also exist; all com pilus systems mediate DNA uptake but may have no other function, whereas T4P have been shown to promote biofilm formation or bacterial aggregation (6, 7), subsurface motility (through twitching) (8, 9), host–cell adhesion (10, 11, 12), and DNA uptake (13, 14), with some systems showing all four functions (6, 8, 15, 16). T4P also have historically been divided into multiple subtypes; type IVa and type IVb, with many recent publications categorizing Tad/Flp pili as within T4P as type IVc pili (17, 18). Some species produce multiple T4P systems with distinct functions. As an example, the *Vibrio cholerae* genome contains three sets of T4P genes, producing Chitin-Regulated T4Pa pili, mannose-sensitive hemagglutinin T4Pa pili, and the T4Pb toxin–coregulated pili (19, 20, 21), each with a distinct function, com for Chitin-Regulated pili, host attachment for mannose-sensitive hemagglutinin pili, and bacterial aggregation for the T4Pb toxin–coregulated pili. The consequences of naturally occurring variation within T4P systems is less well understood.

Variation in T4P subunits could result from diversifying selection with evolutionary pressure being applied by the host adaptive immune system, both antibodies and class II major histocompatibility complex molecules, as well as phage virions that recognize T4P (22, 23, 24, 25, 26). This variation includes the glycosylation of the major pilin subunit in both *Acinetobacter* and *Pseudomonas* (22, 23, 27). In addition to variation in glycosylation, natural variation in the polypeptide sequence of the major pilin of *Pseudomonas aeruginosa* appears to limit the host ranges of T4P-dependent phages (28). Efforts to use the major T4P subunit for vaccine development have shown substantial variation in serology, leading to efforts using subunits from a range of bacterial strains (29) or the creation of chimeric constructs (30).

In the *Acinetobacter baumannii–calcoaceticus* complex (Acb), nonflagellated, Gram-negative coccobacilli associated with multidrug-resistant infections in health care settings, T4P are involved in several distinct physiological processes (natural com, twitching motility, host–cell adhesion, and biofilm formation) and structurally variable (16, 27). Previously, we found structural divergence in the atomic structures of PilA, the major pilin, from three strains of *A. baumannii* (15, 27). We resolved the X-ray crystal structures of PilA from three *A. baumannii* strains, ACICU, AB5075, and BIDMC57. For each of these proteins, the resemblance was closer to a homolog in another species (either *P. aeruginosa* or *Dichelobacter nodosus*) than to either of the other two *A. baumannii* PilA structures (15, 27, 31, 32, 33) (Fig. S1). These three bacterial species, *A. baumannii*, *P. aeruginosa*, *and D. nodosus*, are all members of the family Moraxellaceae and are all opportunistic pathogens of mammals, suggesting that their T4P may be functionally similar.

In an isogenic background, complementation of a *pilA*-null mutant with these three *pilA* genes also resulted in distinct phenotypes for T4P-dependent functions, with +*pilA*^ACICU^ (from the International Clone II [IC-II] group) showing very low twitching motility but higher *in vitro* biofilm formation than +*pilA*^AB5075^ (from the International Clone I [IC-I] group) (15). We attributed these phenotypes to differences in surface electrostatics between the two PilA proteins, which we hypothesized would lead to greater bundling of pili between cells for the IC-II T4P. Pilus bundling between cells would nucleate biofilm, as observed for the bundle-forming pilus (34, 35), but would reduce cellular motility by limiting the ability of cells to move independently of each other. However, observations from other bacterial species show that in some cases bundling of pili from the same cell promotes, and may even be essential for, twitching motility (36, 37, 38).

One potential resolution to this conundrum is to consider DNA uptake, another T4P function that requires retraction, the ability to depolymerize the pilus fiber, restoring the pilin subunits to the inner membrane, and correspondingly shortening the pilus. The *Acinetobacter* T4P system is capable of DNA uptake, the first step in natural com, along with twitching motility, and the induction of biofilm formation and host–cell adherence (Fig. 1). Conceptually, we can divide these four functions into retraction-dependent functions (twitching motility and DNA uptake) and retraction-independent functions (biofilm formation and host–cell adherence), which are purely adhesive and independent of retraction. These differences can be observed in T4P systems through mutations of T4P-associated cytoplasmic AAA + ATPase motors. Extension ATPases (PilB in *A. baumannii*, *Acinetobacter baylyi* also expresses TfpB) are required to polymerize the pilins into the pilus fiber, and *pilB*-null mutants, or cells where PilB is inhibited, show no T4P activity (39, 40). Retraction ATPases (PilT and PilU in *A. baumannii*) depolymerize the pilus fiber, and *pilT*-null mutants show no twitching motility or natural com (16), but the pilT mutant is hyperpiliated (16), and we previously found an increase in host–cell adhesion in a *pilT* mutant (27).

**Figure 1:**
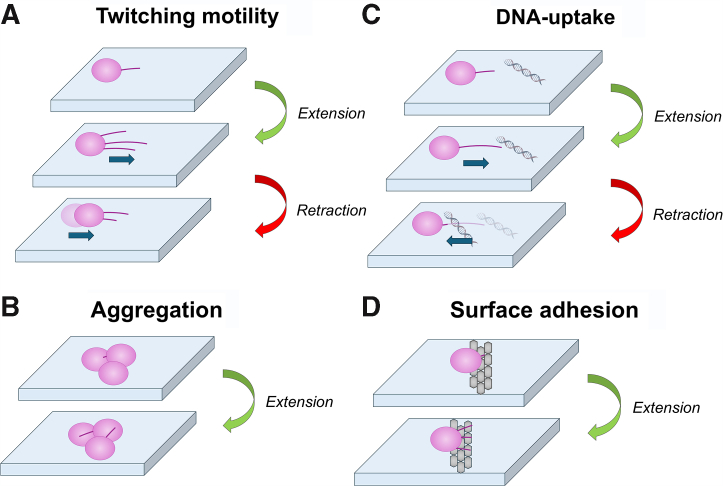
**Retraction-dependent and retraction-independent T4P activities.** Schematic description of four T4P functions. *A,* twitching motility, (*B*) cellular aggregation/biofilm formation, (*C*) DNA uptake (which require the ability to retract pili), and (*D*) surface adhesion (which are independent of retraction). T4P, type IV pili.

Here, we compare retraction-dependent T4P functions, DNA uptake, and twitching motility, as well as retraction-independent biofilm formation in our isogenic *pilA* complement model as well as between naturally occurring IC-I and IC-II *A. baumannii* strains. We found that IC-II T4P, both in an isogenic background and in naturally occurring *A. baumannii* strains, show defects in both twitching motility and DNA uptake but show a greater induction of biofilm when compared with their IC-I counterparts, which may be explained by greater surface T4P expression. Both of these features, loss of retraction-dependent T4P functions and gain of biofilm formation, appear to be the consequences of a natural defect in T4P retraction resulting from the IC-II T4P structure.

## Results

### Differences in surface electrostatics cannot explain differential T4P phenotypes

**Figure 2:**
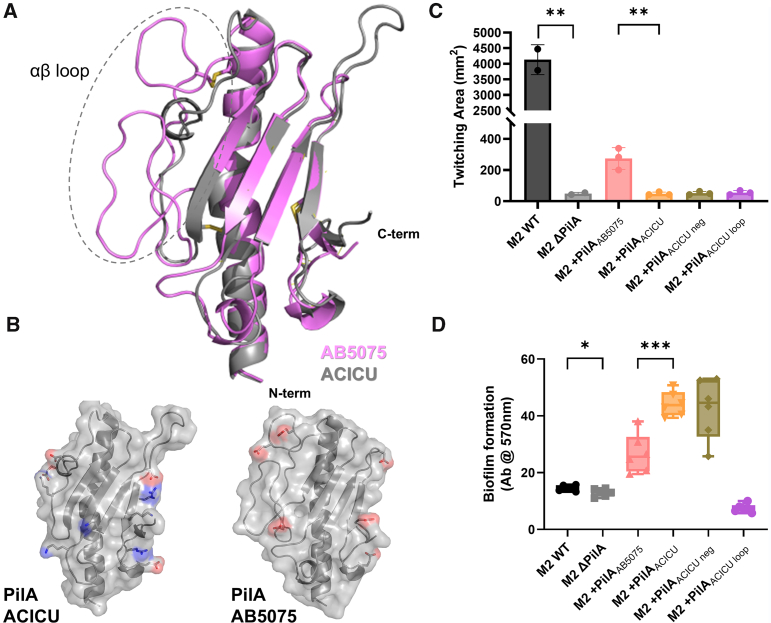
**Twitching motility and biofilm formation for PilA ACICU mutants.***A*, overlay of the PilA^ACICU^ and PilA^AB5075^ structures, AB5075 is in *pink* with ACICU in *gray*, with the αβ-loop encased in a *dashed semicircle*. *B*, surface-charged residues PilA^ACICU^ and PilA^AB5075^ (exposed in a model of the assembled pilus) are depicted either in *red* (acidic, electronegative) or *blue* (basic, electropositive). *C*, twitching motility for M2 wt, Δ*pilA*, and complements. *D*, static biofilm formation for M2 wt, Δ*pilA*, and complements. Significant differences are measured by ANOVA, ∗*p* ≤ 0.05, ∗∗*p* ≤ 0.01, ∗∗∗*p* ≤ 0001, and ∗∗∗∗*p* ≤ 0.0001.

Previously, we found that in this isogenic background, bacteria with AB5075 (IC-I) T4P showed higher twitching motility and lower biofilm formation than those with ACICU (IC-II) T4P (15). We also found that, based on X-ray crystal structures of PilA^ACICU^ and PilA^AB5075^, the AB5075 pilus would have a more electronegative surface area than its ACICU counterpart. We hypothesized a structure–function relationship where T4P bundling between adjacent cells would promote biofilm formation by nucleating assembly and hinder motility by preventing entangled cells from moving independently. We also proposed that the relatively electronegative AB5075 T4P fibers would be less prone to bundling. To test this hypothesis, we made two chimeric mutant *pilA* genes based on structural differences between PilA^ACICU^ and PilA^AB5075^, highlighted in Figure 2, the αβ-loop (*A*), and five surface-exposed basic residues found in PilA^ACICU^ (*B*). Because the electronegativity of the PilA^AB5075^ surface was driven not by an overabundance of acidic residues, but by the absence of basic residues on the surface, we created a *pilA*^ACICU^ gene where each of the surface exposed, basic residues were mutated to the counterpart residues in PilA^AB5075^, K102T, K103T, R122T, K124E, R132N, and *pilA*^ACICU-negative^ (Fig. S3). Because the greatest concentration of structural differences between the two PilA proteins is in the αβ-loop, we also created a mutant *pilA*^ACICU^ gene where the sequence of the PilA^AB5075^ αβ-loop (residues 54–93) was substituted for the PilA^ACICU^ αβ-loop (residues 54–78), *pilA*^ACICU-loop^.

However, as the results of functional comparisons show in Figure 2, C and D, neither of these alterations resulted in the high-motility, low-biofilm phenotype seen from PilA^AB5075^. Complementation with *pilA*^ACICU-loop^ resulted in a *pilA*-null phenotype for both twitching motility and biofilm formation (Fig. 2), suggesting that the chimeric protein was unable to fold efficiently. The *pilA*^ACICU-negative^ complement produced results identical to *pilA*^ACICU^ in twitching motility (Fig. 2*C*) and biofilm formation (Fig. 2*D*). These results suggest that the difference between the AB5075 and ACICU T4P phenotypes is not a product of differential surface electrostatics but some other differential aspects of the two pilus structures.

### In an isogenic background, IC-II T4P show decreased com compared with IC-I T4P

**Figure 3:**
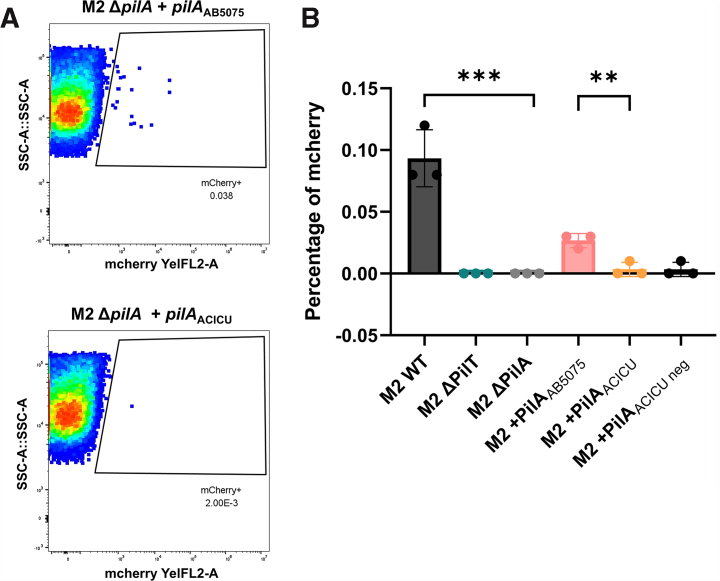
**DNA uptake by IC-I and IC-II T4P in an isogenic background.***A*, representative data from DNA uptake measurements (of an mCherry-encoding plasmid) by flow cytometry for *Acinetobacter nosocomialis* M2 Δ*pilA* + *pilA*^AB5075^ (IC-I) and *A. nosocomialis* M2 Δ*pilA* + *pilA*^ACICU^ (IC-II). *B*, DNA uptake for M2 wt, mutants, and complements. Significant differences are measured by ANOVA, ∗*p* ≤ 0.05, ∗∗*p* ≤ 0.01, ∗∗∗*p* ≤ 0001, and ∗∗∗∗*p* ≤ 0.0001. IC-I, International Clone I; IC-II, International Clone II.

Natural com or natural transformation, the ability to take up, internalize, and replicate extracellular DNA, is directly reliant on DNA uptake, and previously, mutants of the T4P system abrogating either pilus biogenesis (41), pilus retraction (16), or DNA binding (42) have been shown to have defects in natural com. If we attributed the motility defect of the +*pilA*^ACICU^ complement to a defect in T4P retraction, we would expect to see a defect in DNA uptake as well. To compare the relative ability of IC-I and IC-II T4P systems to take up extracellular DNA, we used isogenic chimeric strains, complementing a *ΔpilA* mutant of a genetically tractable model organism, *Acinetobacter nosocomialis* M2 (originally described as *A. baumannii* M2) (16). Each of the constructed strains expresses *pilA* from strains AB5075 (IC-I) or ACICU (IC-II) using the pUCP20GM plasmid as previously described (15). All strains were grown to the exponential phase and incubated with an mCherry plasmid in broth. Uptake of the plasmid was quantified through red fluorescence for each strain using flow cytometry as described above, with methods adapted from Godeux *et al.* (43). Results from this experiment are shown in Figure 3 with *A* showing representative data from the +*pilA*^AB5075^ and +*pilA*^ACICU^ complemented strains, whereas *B* shows the complete cross-strain comparison. As expected, WT *A. nosocomialis* M2 bacteria are capable of natural com under these conditions, but Δ*pilA* and Δ*pilT* strains, which are deficient in retraction (Δ*pilT*) or pilus biogenesis (Δ*pilA*), showed no natural com. For the complemented strains, we found that the AB5075 complement was competent, but the ACICU complement was significantly less able to take up DNA (*p* = 0.0078), statistically indistinguishable from the Δ*pilA* strain it was derived from (*p* = 0.3739). As we observed for twitching motility, the surface-electrostatic mutant + *pilA*^ACICU-negative^ was functionally identical to +*pilA*^ACICU^. The use of flow cytometry did not require the induction of antibiotic resistance to measure com, but similar results were obtained by measuring the induction of antibiotic resistance through the uptake of linearized plasmid (Fig. S4) (16).

### IC-II strains show defects in retraction-dependent T4P activity

Based on our findings for +*pilA*^ACICU^ and +*pilA*^AB5075^ complements in the M2 background, we hypothesized that the structural differences in IC-I and IC-II PilA proteins were responsible for the differential phenotypes for T4P functions. However, a defect in pilus retraction observed in a chimeric +*pilA*^ACICU^ cell could potentially be an artifact not fully representative of naturally occurring strains expressing ACICU-like PilA proteins. The *pilA* gene is more variable in *Acinetobacter* than other T4P genes (15, 27), but differences in other genes, including other pilin subunits or pilus biogenesis genes, could also impact T4P phenotypes. Prior studies by other groups have shown differences between IC-I and IC-II *A. baumannii* strains in surface behavior, particularly twitching motility (44, 45), generally in qualitative assays. To quantitatively compare retraction-dependent functions, twitching motility and DNA uptake, and biofilm formation (which T4P promote without retraction (46)), we used naturally occurring *A. baumannii* strains expressing PilA protein variants.

**Figure 4:**
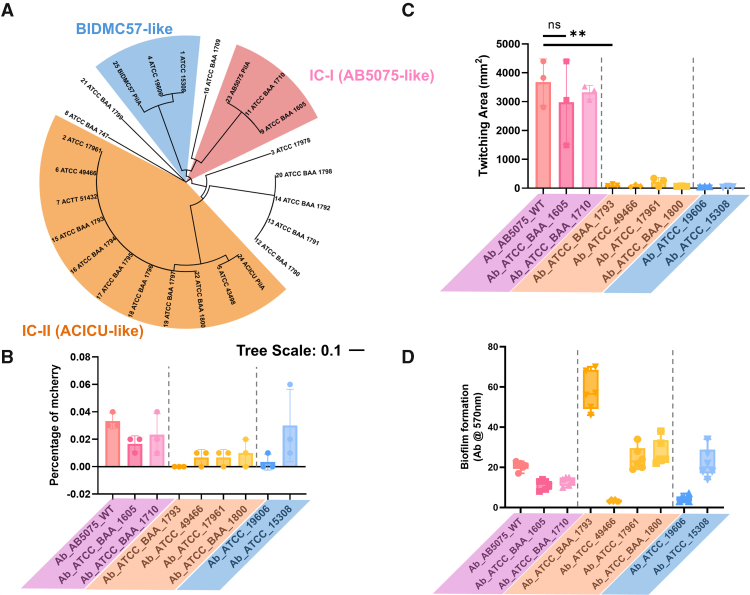
**T4P-dependent function in IC-I and IC-II *Acinetobacter baumannii* strains.***A*, dendrogram of *A. baumannii* strains by PilA sequence, clusters are highlighted corresponding to PilA^ACICU^ (*orange*), PilA^AB5075^ (*pink*), and PilA^BIDMC57^ (*blue*). *B*, DNA uptake of an mCherry plasmid by flow cytometry; strains are colored by PilA subtype with the IC-I (AB5075) cluster in *pink*, the IC-II (ACICU) cluster in *yellow*, and the BIDMC-57 cluster in *blue*. *C*, twitching motility with identical coloration to *B*. *D*, static biofilm formation measured by crystal violet staining with strains colored identically to *B* and *C*. Significant differences are measured by ANOVA, ∗*p* ≤ 0.05, ∗∗*p* ≤ 0.01, ∗∗∗*p* ≤ 0001, and ∗∗∗∗*p* ≤ 0.0001. IC-I, International Clone I; IC-II, International Clone II; T4P, type IV pili.

Previously, our group divided *A. baumannii* PilA into three subtypes based on sequence analysis and our resolution of the X-ray crystal structures of PilA from three *A. baumannii* strains, AB5075 (IC-I), ACICU (IC-II), and BIDMC-57 (fitting into neither of the IC groups and with PilA sequences similar to the model system *A. nosocomialis* M2 and strain *A. baumannii* American Type Culture Collection [ATCC] 19606) (15, 27). Although the overall conservation for *A. baumannii* PilA is quite low (PilA^AB5075^ and PilA^ACICU^ are ∼40% identical), conservation within the three proposed subtypes is over 90% (Fig. S5). To quantitatively compare T4P functions in naturally occurring strains, we obtained eight *A. baumannii* strains from the ATCC, including two with AB5075-like (IC-I) *pilA* genes, four with ACICU-like (IC-II) *pilA* genes, and two with BIDMC-57-like PilA sequences. These strains (along with *A. baumanii* AB5075) were assayed for three T4P-dependent functions, twitching motility (*B*), DNA uptake (*C*), and biofilm formation (*D*) as shown in Figure 4.

All four IC-II isolates were defective for retraction, as evidenced by a lack of both DNA uptake and motility. In contrast, the IC-I strains all showed robust twitching motility and DNA uptake. The two isolates from the third group (those with BIDMC-57-like PilA proteins) were nonmotile, but ATCC 15308 showed robust DNA uptake and modest biofilm formation, similar to the IC-I strains. On average, IC-II isolates formed more biofilm than IC-I isolates, but ATCC 49466 showed a defect for biofilm formation, similar to the *pilA* mutant. We hypothesize that ATCC 49466, along with ATCC 19606, are largely unable to generate T4P under these experimental conditions, as they were negative for all three functions. Because *A. baumannii* ATCC 15308 and ATCC 19606 have identical *pilA* sequences, as do ATCC 49466 and ATCC 17961 (Fig. S5), these functional defects must stem from differences in other pilus genes or the regulation of pilus function rather than the T4P themselves. This observation is consistent with negative results for ATCC 19606 twitching motility observed by others (44, 47) despite the fact that PilA in *A. baumannii* ATCC 19606 and PilA in *A. nosocomialis* M2 are 92% identical (Fig. S2) and M2 shows twitching motility under identical conditions (Fig. 3*C*).

To evaluate the impact of retraction deficiency on biofilm formation, we measured static biofilm formation for this panel of nine naturally occurring *A. baumannii* strains. As expected, IC-II (ACICU-like) strains generally showed enhanced biofilm formation compared with IC-I (AB5075-like) strains (Fig. 4*D*). Notably, despite belonging to IC-II, *A. baumannii* ATCC 49466 exhibited reduced biofilm formation (along with poor twitching motility and DNA uptake), likely because of incompatibility with the growth conditions used in these assays, similar to the results described above for *A. baumannii* ATCC 19606. These findings suggest that biofilm formation does not require T4P retraction, and in fact, retraction deficiency promotes biofilm formation in *A. baumannii*.

### Retraction deficiency induces biofilm formation in *A. baumannii*

**Figure 5:**
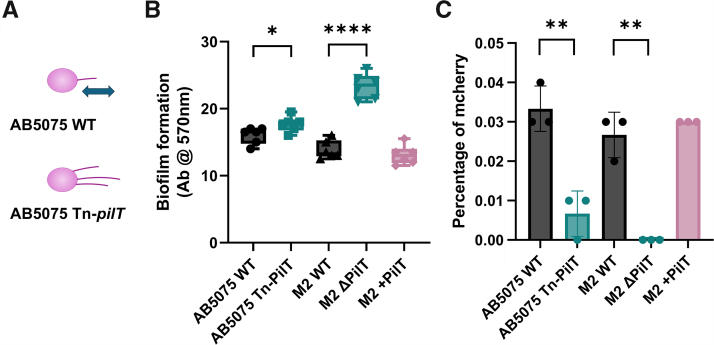
**DNA uptake and biofilm formation by *pilT* mutants.***A*, schematic representation of *Acinetobacter baumannii* WT and Δ*pilT* showing the retraction defect and increase in piliation for Δ*pilT*. *B*, static biofilm formation for *A. nosocomialis* M2 and *A. baumannii* AB5075 *pilT* mutants. *C*, DNA uptake by flow cytometry for *pilT* mutants. Significant differences are measured by ANOVA, ∗*p* ≤ 0.05, ∗∗*p* ≤ 0.01, ∗∗∗*p* ≤ 0001, and ∗∗∗∗*p* ≤ 0.0001.

To test the hypothesis that retraction deficiency promotes retraction-independent T4P functions, including biofilm formation, we measured static biofilm formation in *A. nosocomialis* M2 and *A. baumannii* AB5075 for the WT strains and *pilT* mutants (M2 Δ*pilT* and AB5075 Tn-*pilT*-). Prior studies in multiple organisms have shown that *pilT* is required for efficient retraction of T4P, as evidenced by DNA uptake/natural transformation and macroscopic twitching motility (11, 16, 48). Our group previously found that an M2 *pilT* mutant showed a defect in twitching motility but showed significantly greater adherence to A549 and Detroit 562 monolayers (15). Figure 5 shows the results of assays of biofilm formation (*B*) and DNA uptake (*C*). Regardless of genetic background, the *pilT* mutants showed defects in DNA uptake but an induction of static biofilm compared with the parent strains. Unlike comparisons between IC-I and IC-II *A. baumannii* strains or the *pilA*^AB5075^ and *pilA*^ACICU^ complements of M2 Δ*pilA*, these effects can be ascribed solely to defects in retraction, as no modification was made to the *pilA* genes. We hypothesize that this induction in biofilm formation is driven by an increase in surface piliation (46), consistent with results from the isolation of sheared pili from the surface of *A. baumannii* cells as described below.

### Complementation with IC-II T4P leads to greater surface expression of T4P

If defects in retraction can explain the greater induction of *in vitro* biofilm formation shown by *pilT* mutants and IC-II T4P systems, we would expect both to be products of greater surface expression of T4P, either in the number of pili per cell or through longer pili. In a comparison of transmission electron microscopy (TEM) images, greater numbers of pili were observed previously in *A. nosocomialis* for a *pilT* mutant (16), but unambiguously differentiating T4P and chaperone-usher (CU) pili is difficult at these resolutions because of their similar width, 6 to 8 nm (23).

**Figure 6:**
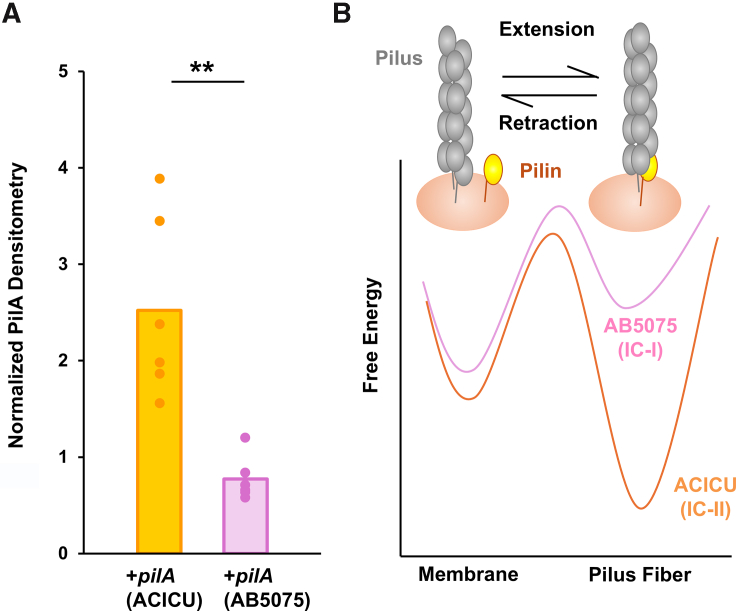
**T4P surface expression.***A*, normalized PilA surface expression for *Acinetobacter nosocomialis* M2 Δ*pilA* + *pilA*^AB5075^ (IC-I) and *A. nosocomialis* M2 Δ*pilA* + *pilA*^ACICU^ (IC-II) as measured by densitometry of a PilA band using silver staining. *B*, free energy diagram showing energetic barriers for T4P retraction and the putative differences between AB5075 and ACICU T4P. Significant differences are measured from the WT strain using the Student’s one-tailed *t* test, ∗*p* ≤ 0.05, ∗∗*p* ≤ 0.01, ∗∗∗*p* ≤ 0001, and ∗∗∗∗*p* ≤ 0.0001. IC-II, International Clone II; T4P, type IV pili.

To compare T4P surface expression between the IC-I and IC-II subtypes, we used growth conditions expected to favor the production of T4P over CU pili, selective precipitation to reduce levels of contaminants, and compared levels of surface PilA by gel electrophoresis and densitometry. Comparisons of *A. baumannii* AB5075 WT and Tn-*pilT*- showed greater surface PilA expression for the *pilT* mutant (Fig. S6). We compared *A. nosocomialis* M2 Δ*pilA* + *pilA*^ACICU^ and M2 Δ*pilA* + *pilA*^AB5075^, using the parent strain, M2 Δ*pilA* as a negative control to ensure that we could unambiguously assign the PilA band (Fig. S7). The results of this experiment are shown in Figure 6*A*, where complementation with the IC-II subtype (*pilA*^ACICU^) resulted in significantly higher levels of PilA sheared from the surface per bacterial cell.

### Isolated IC-II T4P show greater thermal stability than IC-I T4P

**Figure 7:**
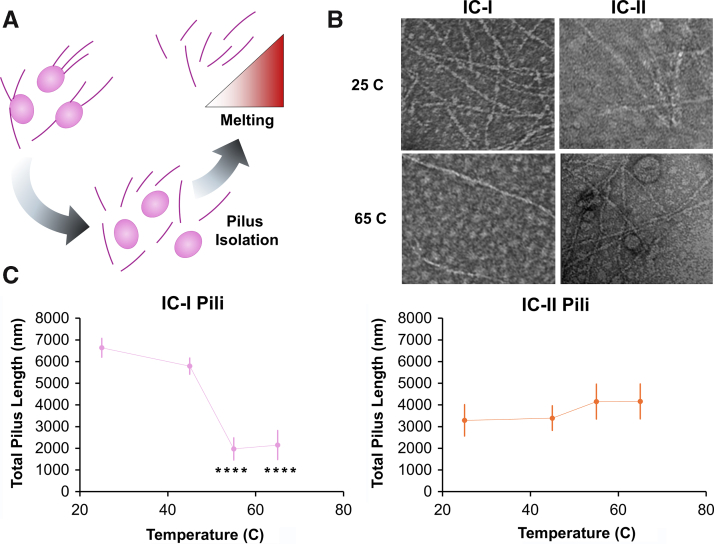
**Stability of IC-I and IC-II T4P.***A*, pilus isolation schematic. *B*, representative TEM images of +pilA^AB5075^ (IC-I) and +pilA^ACICU^ (IC-II) pili at 25° C and 65° C. *C*, average total pilus length per field (n = 4) as a function of temperature for IC-I (*left*) and IC-II (*right*) pili. Error bars mark one standard deviation. Significant differences are measured from 25° C using the Student’s one-tailed *t* test, ∗*p* ≤ 0.05, ∗∗*p* ≤ 0.01, ∗∗∗*p* ≤ 0001, and ∗∗∗∗*p* ≤ 0.0001. IC-I, International Clone I; IC-II, International Clone II; TEM, transmission electron microscopy; T4P, type IV pili.

If T4P from IC-I strains are more difficult to retract because of differences in the free energy change of pilus formation, as we hypothesize in Figure 6, these differences should be evident in differences in the stability of pili isolated from bacterial cells. Previously, our group has used thermal denaturation to observe the stability of the folded state of pilin proteins using circular dichroism (27), but observing the transition from assembled pili into dissembled subunits presents several difficulties for this method. More recently, Chlebek *et al.* (49) showed that differences in pilus stability can be measured using a combination of thermal denaturation and fluorescence microscopy. Building upon this method, we sheared the surface of *A. nosocomialis* M2 *ΔpilA* + *pilA*^AB5075^ (IC-I) and + *pilA*^ACICU^ (IC-II) and isolated pili from these distinct structural subtypes as described in the Experimental procedures section. These samples were incubated at temperatures ranging from 25 °C to 65 °C for 1 h, fixed, negatively stained, and observed by TEM as shown in Figure 7, *A* and *B*.

The total pilus length for each field was measured by tracing by hand, and we observed a greater average length of pili per field in the +*pilA*^AB5075^ sample for the first two temperature points (consistent with our observation that growth for this strain on solid media is more robust), which rapidly decreased by 55 °C resulting a statistically significant (*p* ≤ 0.0001) drop in the total pilus length per field. By contrast, the +*pilA*^ACICU^ samples showed no significant differences in total pilus length over the observed temperature range (*C*).

## Discussion

The type IV filament structure is unique in its reversible assembly. Unlike CU or sortase pilus systems, type IV pilins can be polymerized into pili and pili depolymerized into pilins through reversed rotations of cytoplasmic ATPase motors (PilB, PilT, and PilU) attached to the same biogenesis machinery (PilC, PilM, PilN, PilO, and PilP). Pilus retraction provides unique benefits for T4P systems by enabling twitching motility and DNA uptake, which, in *Acinetobacter*, are absolutely dependent upon T4P function. This stands in contrast to retraction-independent T4P adhesive activity, promoting host cell adhesion and biofilm formation, which occur through multiple molecular mechanisms.

Despite the obvious benefits of efficient pilus retraction, data from retraction ATPase mutants indicate that it imposes costs as well. Mutants of *pilT* in *P. aeruginosa* were observed previously to be hyperpiliated (46), and Harding *et al.* (16) were able to observe T4P on the surface of an *A. nosocomialis* M2 Δ*pilT* mutant but not the parent strain. To the extent to which T4P act as adhesins, reductions in their number will result in decreased adhesion (and *vice versa*), and indeed, we show here that *Acinetobacter pilT* mutants show an induction of biofilm compared with their parent strains.

If pilus retraction provides novel functions for T4P and also restricts adhesive functions, this implies that (i) T4P retraction should be viewed as a scalable quality and that an optimum likely exists between the most extreme possible cases; pili being incapable of retracting from the cell surface or retraction outstripping extension to completely remove T4P from the cell surface and (ii) that the optimum level of retraction is likely to vary depending on environmental conditions and for specific T4P functions.

As we have noted previously, the *pilA* gene in *Acinetobacter* is more variable, not only than the pilus processing/extension/retraction machinery (PilBCD, PilMNOP, etc) but also other pilin genes (15, 27). Variation in surface-exposed residues may be driven by diversifying selection (50), but the variation in *Acinetobacter* PilA is not limited to surface-exposed residues but includes substantial differences in regions likely to impact pilus assembly. Taken together, these features imply that variation in *pilA* may be a critical mechanism for modulating T4P functions, not only in *A. baumannii* but also in related species, which are found both in the environment and as opportunistic pathogens and where the major T4P subunit shows similar variation, including *P. aeruginosa* and *D. nodosus*. However, we note that *P. aeruginosa* strains with PilA subunits structurally similar to PilA^ACICU^, PAO1, and PAK are more motile than *A. baumannii* IC-II strains (51, 52), suggesting that other factors may also impact the balance between extension and retraction.

Previously, Chlebek *et al.* (49) identified pilus-stabilizing interactions as a key factor in motor-independent, spontaneous retraction, where differences could be observed based on specific single amino acid mutations. The target amino acids, found at the interfaces between pilus subunits, were identified as suppressors of the natural transformation defect of a Δ*pilT* mutant, and the mutations converted these generally bulky residues to glycines, destabilizing the pilus fiber. These differences were only observed through an increase in spontaneous retraction independent of the retraction ATPase; no differences were observed for single mutations in conventional motor-dependent retraction.

Here, we show that naturally occurring variation in T4P structure leads to significant differences in motor-mediated pilus retraction, with consequences for retraction-dependent and retraction-independent T4P functions. Contrary to our expectations, variation in surface-exposed residues between two T4P subtypes, PilA^ACICU^ and PilA^AB5075^, has no direct impact on the functional differences we observed both in an isogenic background and in the naturally occurring IC-I and IC-II strains. This is evidenced by the commonality of phenotypes between the M2 Δ*pilA* + *pilA*^ACICU^, *pilA*^ACICU_negative^, and naturally occurring IC-II bacteria. Instead, differences between the two are driven by a combination of decreased pilus retraction and increased surface piliation for the IC-II T4P system, both of which could be explained by increased pilus stability creating an energetic barrier to pilus retraction.

Clinical epidemiology also suggests that *Acinetobacter* isolates vary in T4P-mediated surface behavior based on their origin, with lung isolates showing, in aggregate, greater biofilm formation and blood isolates greater twitching motility (53). We hypothesize that these differences stem from the distribution of T4P subtypes within these populations. In a survey of *Acinetobacter* infections in Japan, Matsui *et al.* (54) found that IC-II strains accounted for 43% of *A. baumannii* respiratory infections but none of the blood infections and only 2% of all other infections. Comparing surface behavior from different *A. baumannii* isolates, Eijkelkamp *et al.* (44) characterized only 9% of IC-II isolates as positive for twitching motility, but 100% of IC-I isolates were positive. Conversely, median biofilm formation was found to be approximately 70% greater for IC-II isolates. Skerniškytė *et al.* (45) also found greater twitching (but not swarming) motility for IC-I strains compared with IC-II and greater desiccation resistance for IC-II strains. Vijayakumar *et al.* (53) also found that *A. baumannii* isolates varied considerably in their surface behavior based on the site of isolation. Their results showed that respiratory isolates formed more biofilm but were less motile in twitching assays than blood isolates. We believe that all these results can be explained through differences in pilus retraction similar to those observed here.

Despite the strong association in the literature between T4P and twitching motility (16, 36, 38, 42, 48, 55, 56, 57, 58, 59, 60, 61), the evolution of retraction-deficient T4P systems in *Acinetobacter* is plausible not only because of the potential advantages for adhesive functions but also because pilus retraction may be more useful in the environment than in a host lifecycle and may carry associated risks. Although mutants of *pilA* have been found to be less pathogenic in an animal model (62), twitching motility itself appears to be less important than surface motility *in vivo* (63), and IC-II strains with poor motility are still virulent in murine infections (45). Pilus retraction also carries risks in terms of phage infection, as retraction is an essential step in T4P-mediated phage infection, and *pilT* mutants are immune (22, 24).

If *A. baumannii* T4P systems are, in fact, specialized for distinct functions, that specialization must stem from measurable physical differences between some component(s) of pilus itself or the apparatus responsible for pilus extension and retraction. Here, we show that we can reproduce the phenotypic differences found in naturally occurring IC-I and IC-II strains in an isogenic background solely through modifications in *pilA*. In *A. baumannii*, there are over a dozen gene products necessary for pilus biogenesis and function, including seven putative pilin subunits, but it is nevertheless unsurprising that PilA, which makes up the vast majority of the pilus, has a controlling role. Because the vast majority of the subunits in a T4P are PilA, interactions between the pilus machinery during extension and retraction are primarily with PilA, and interactions between PilA subunits are the principal stabilizing force of pilus assembly. In Figure 6*B*, we present a model by which an increase in stabilizing interactions between assembled PilA subunits could create an energetic barrier to retraction, which decreases retraction speed by opposing the force of the PilT motor. We believe this model represents the best current explanation for the phenotypic differences in T4P function, both because, as we have shown here, these differences can be reproduced solely through variation in *pilA* in an isogenic background, and because complementation with an IC-II *pilA* gene produces relative defects in both retraction-mediated T4P functions when compared with an IC-I *pilA* complement.

Taken together, the results here suggest that the evolution of pilus retraction is a double-edged sword, with the addition of new capabilities, twitching motility and DNA uptake, balanced against a reduction in adhesive capability. This balancing act may favor different pilus structures with different levels of potential retraction depending on the conditions in the host or other environment and suggests that variation on the structures of T4P subunits within bacterial species is partially a product of lifecycle adaptation.

## Experimental procedures

### Strains and plasmids

Details on *A. nosocomialis* M2 and mutants can be found here (16) (where it was originally identified as *A. baumannii* M2). *A. baumannii* AB5075 WT and mutants are from the University of Washington AB5075-UW transposon mutant library maintained by the Manoil group (64). Strains from the IC-I and IC-II groups were provided by the ATCC. Details of complementation *by pilA* genes from *A. baumannii* AB5075, ACICU, and BIDMC57 can be found in the study by Ronish *et al.* (15). The *pilA* ACICU negative and αβ-loop mutants (sequences available in Table S1) were synthesized by GenScript and ligated into pUCP20GM (46) using BamH1 and HindIII restriction sites. The resulting vectors were electroporated into *A. nosocomialis* M2 Δ*pilA* (16) using standard protocols (65). The presence of the plasmids was confirmed by both resistance to gentamycin and PCR of the pilin genes. For plasmid maintenance, strains complemented with *pilA*::pUCP20GM were grown on solid media supplemented with 50 μg/ml gentamycin before their use in the assays described later.

### Twitching motility

Macroscopic twitching motility was assessed using standard protocols (15, 27, 66). Briefly, *Acinetobacter* strains, including mutants and complemented strains, were grown on 1.5% MacConkey agar plates overnight. Colonies were selected and stabbed through the centers of 1% MacConkey agar plates in polystyrene petri dishes. The plates were incubated in sealed bags at 37 °C for 3 days. The agar was then removed, and the bacteria adhered to the polystyrene Petri dish stained with 0.1% crystal violet for 5 min. Excess crystal violet was removed by gentle washing with deionized water three times. The subsurface twitching area on each plate was accessed by measuring the diameters of the ellipse and then calculating the area (67). Statistics were calculated for three replicates, and significance was determined by the Student’s *t* test.

### Static biofilm formation

Static biofilm formation was quantified using horizontal growth on 6-well plates, with frequent changes of media and quantification through staining with crystal violet. Briefly, *Acinetobacter* strains, including mutants and complemented strains, were grown in LB no salt broth (tryptone 10 g/l, yeast extract 5 g/l) overnight. The bacteria were inoculated into 6-well plates to have a final absorbance of 0.1 at 600 nm in 5 ml total volume. The 6-well plates were incubated at 37 °C for 3 days (72 h). To remove nonadherent, planktonic bacteria and introduce fresh growth media, the medium was changed every 20 to 24 h using a pipette. After 72 h, 5 ml of PBS was added to and removed from the plates to wash the planktonic bacteria. Five milliliters of 0.1% crystal violet with 2.5% glutaraldehyde was added to the plates and stained for 10 min. Excess crystal violet was removed by gentle washing with PBS. One milliliter of 100% ethanol was then added to the plates to dissolve the dye. Relative biomass was assessed using the final concentration of released crystal violet; the absorbance value was read using a NanoDrop-C at 570 nm.

### DNA uptake through flow cytometry

Uptake of extracellular DNA was quantified using flow cytometry to measure the proportion of fluorescent bacterial cells after incubation with a plasmid encoding mCherry. This method is adapted from the study by Godeux *et al.* (43). Briefly, *Acinetobacter* strains, including mutants and complemented strains, were grown in LB no salt broth overnight. The cultures were then diluted 1:200 into 200 μl LB no salt broth, and 500 ng of plasmid encoding mCherry was added. The broth was then grown at 37 °C for 4 h. Bacterial suspensions were fixed with formaldehyde (final concentration, 3.7%) for 30 min at room temperature and then washed twice with PBS and further resuspended in 200 μl PBS. Flow cytometry was performed using a Beckman Coulter CytoFLEX LX. Samples were run at a collection rate of 60 μl/min, the mCherry was excited by the 561 nm laser, and fluorescence emission was detected using a 610/20 band pass filter. Data were acquired using CytExpert software (Beckman Coulter) and further analyzed in FlowJo (BD Biosciences).

### Quantifying pilus expression

Pilin proteins were isolated using the methods of Voisin *et al.* (68) with modifications. Bacteria were spread on 10 LB no salt agar plates and grown at 30 °C overnight. Next day, the bacteria were gently scraped from each plate using a sterile cell scraper and resuspended in a 3 ml buffer containing 50 mM Tris–HCl and 150 mM NaCl (pH 8.3). The cells were injected through a needle (diameter 18G 1” blunt needle+ 25G 1”) three times to shear off the pili. The cell suspension was then spun down for 5 min at 13,300*g* at 4 °C. The supernatant was transferred to new 1.5 ml microcentrifuge tubes and centrifuged for an additional 25 min for the same speed at 4 °C. For precipitating the sheared pili, 1 M MgCl_2_ was added to the supernatant to a final concentration of 0.1 M, and the samples were incubated at 4 °C overnight. Samples were further centrifuged for 25 min at 13,300*g* at 4 °C. Then the pellet was resuspended in 100 μl of buffer (50 mM Tris–HCl, 150 mM NaCl, pH 8.3) and stored at 4 °C. Relative PilA surface expression was compared using densitometry by ImageJ from silver-stained SDS-PAGE gels (67); with relative expression calculated by comparing the PilA band to a control band found in both WT and Δ*pilA* samples.

### Measuring pilus stability as a function of temperature

T4P were isolated from *A. nosocomialis* M2 *ΔpilA* + *pilA*^AB5075^ (IC-I) and *A. nosocomialis* M2 *ΔpilA* + *pilA*^ACICU^ (IC-II) as described previously. Resuspended pili were incubated for 1 h at either 25 °C, 45 °C, 55 °C, or 65 °C, fixed by adding glutaraldehyde to a final concentration of 2.5%, pipetted onto 200-mesh carbon formvar–coated copper grids, stained with 1% uranyl acetate, and examined at 80 kV using a Hitachi HT-7800 TEM. The length of intact pili in each field was quantified by tracing pili using the GNU Image Manipulation Program, and this analysis was performed in duplicate independently by K. H. P. and R. M., who reported similar values.

### Figures and diagrams

Data figures were created using GraphPad Prism (GraphPad Software). Images of the PilA^ACICU^ (Protein Data Bank ID: 4XA2) and PilA^AB5075^ (Protein Data Bank ID: 5VAW) X-ray crystal structures were made using UCSF ChimeraX (69), and the dendrogram of PilA sequences was created using Interactive Tree of Life (70).

### Statistical analysis

All statistical analyses were performed using GraphPad Prism, version 9.5.0 (GraphPad Software). All significant differences were determined at *p* < 0.05. Relevant statistical tests used for comparisons are discussed in the figure captions.

## Data availability

The data used in this study are included in this article apart from raw image files used to calculate twitching motility, DNA uptake, or average pilus length. Those images are available from the corresponding author upon request.

## Supporting information

This article contains supporting information (16, 71).

## Conflict of interest

The authors declare that they have no conflicts of interest with the contents of this article.
