# Supplementary material for: Variation in type IV pilus stability modulates DNA uptake and biofilm formation
Source: J Biol Chem. 2025 Oct 6;301(11):110787. doi: 10.1016/j.jbc.2025.110787 (PMC12603735; doi:10.1016/j.jbc.2025.110787)
Supplement: Supplemental Material 2 [file mmc1.docx]

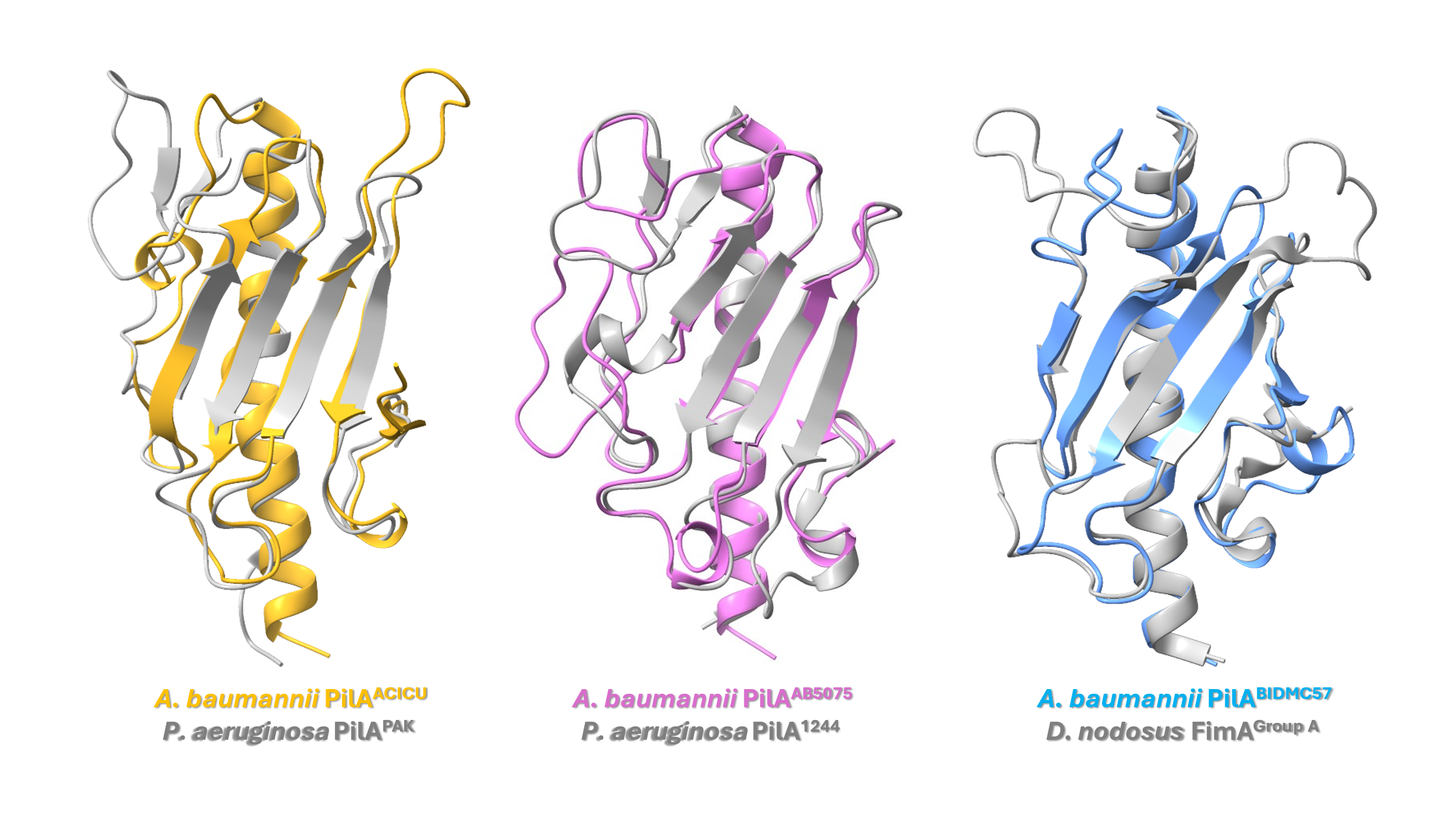


**Supplementary Figure 1:** Superimpositions of the X-ray crystal structures of pilin C-terminal domains. The structures of three *Acinetobacter baumannii* PilA proteins, PilA^ACICU^ (gold), PilA^AB5075^ (magenta) and PilA^BIDMC57^ (blue) are superimposed on structural similar pilin protein homologues from (respectively) *P. aeruginosa* K, *P. aeruginosa* 1244 and group A *D. nodosus*.

**CLUSTAL O(1.2.4) multiple sequence alignment**

**ATCC_19606 FTLIELMIVVAIIGILAAIAIPAYQNYIAKSQASEAFTLADGLKTTINTNLQAGTCFAGG 60**

**M2 FTLIELMIVVAIIGILAAIAIPAYQNYIAKSQASEAFTLADGLKTTINTNLQAGTCFAGG 60**

****************************************************************

**ATCC_19606 ATAATAADQVAGKYGDAEIGGTAPNCTITYTFKSSGVSTKLTSKQIVMNVSETGILTKNS 120**

**M2 ATAVTAADKVSGKYGDAEIGGTAPNCTITYTFKSSGVSNKLTSTKIVMNVSETGILTKNS 120**

*****.****:*:***************************.****.:*****************

**ATCC_19606 STNAPAELLPQSFTAS 136**

**M2 GTDTPVELLPQSFVAS 136**

**.*::*.*******.****

**Supplementary Figure 2:** Clustal Omega alignment of mature PilA amino acid sequences from *Acinetobacter baumannii* ATCC 19606 (top) and *Acinetobacter nosocomialis* M2 (bottom)

**Supplementary Figure 3:** Natural competence by pilus subtype. CFU of streptomycin-resistant bacteria are shown for a natural competence experiment where complements of *Acinetobacter nosocomialis* M2 Δ*pilA* with *pilA*^ACICU^, *pilA*^AB5075^ and *pilA*^BIDMC57^. Natural competence was measured using standard methods (*1*). Briefly, bacterial broth cultures (in MacConkey medium) were mixed with a streptomycin-resistance plasmid and plated on MacConkey agar without antibiotics. After a one hour incubation at 37ºC, the bacteria were scraped, resuspended and replated (with sequential 1:10 dilutions) on MacConkey medium with 50ug/ml streptomycin.


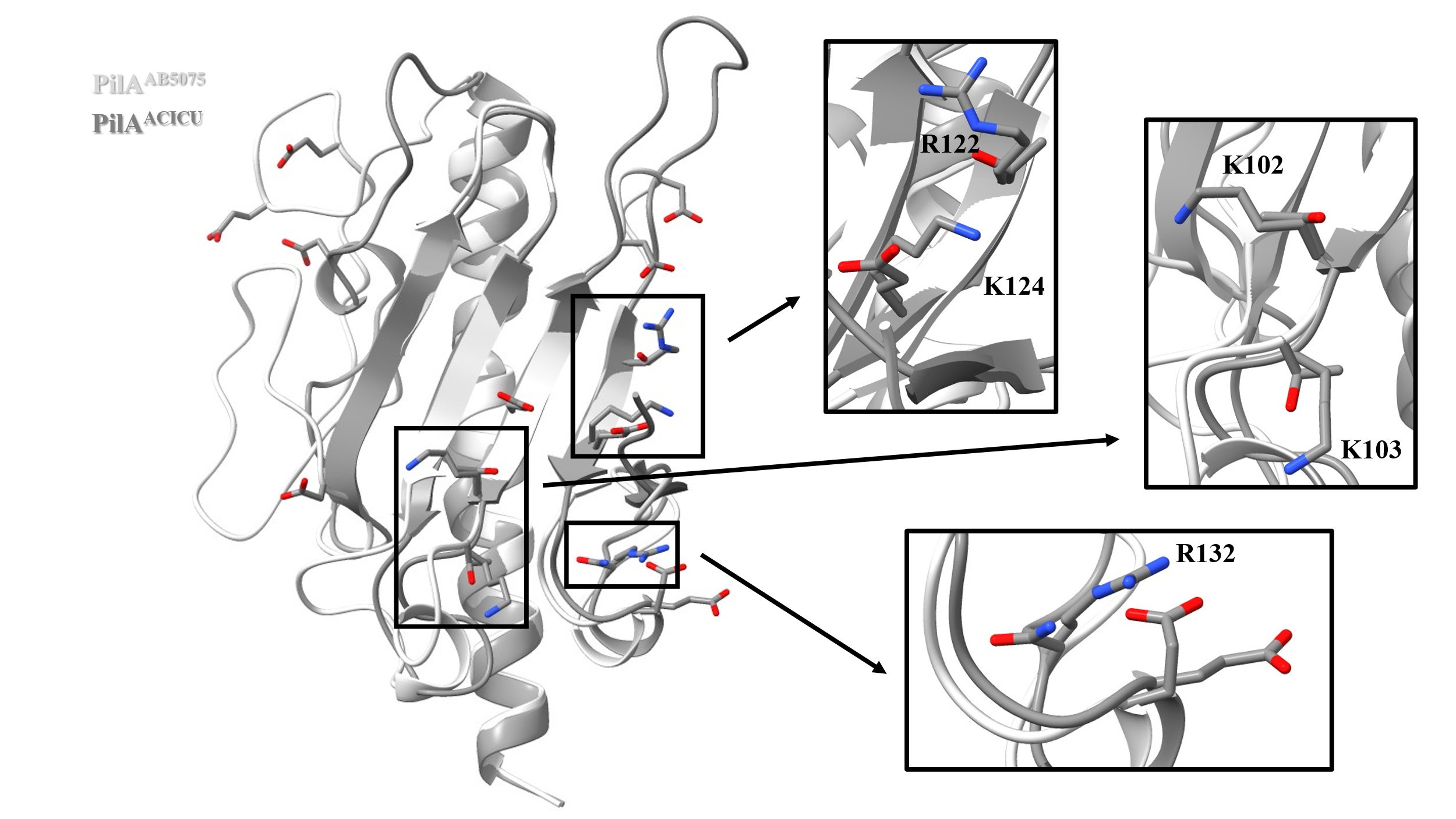


**Supplementary Figure 4:** Single amino acid mutations for the creation of the *pilA*^ACICU^-neg construct. Basic residues from PilA^ACICU^ (dark grey) were mutated to their equivalents from PilA^AB5075^ (light grey).


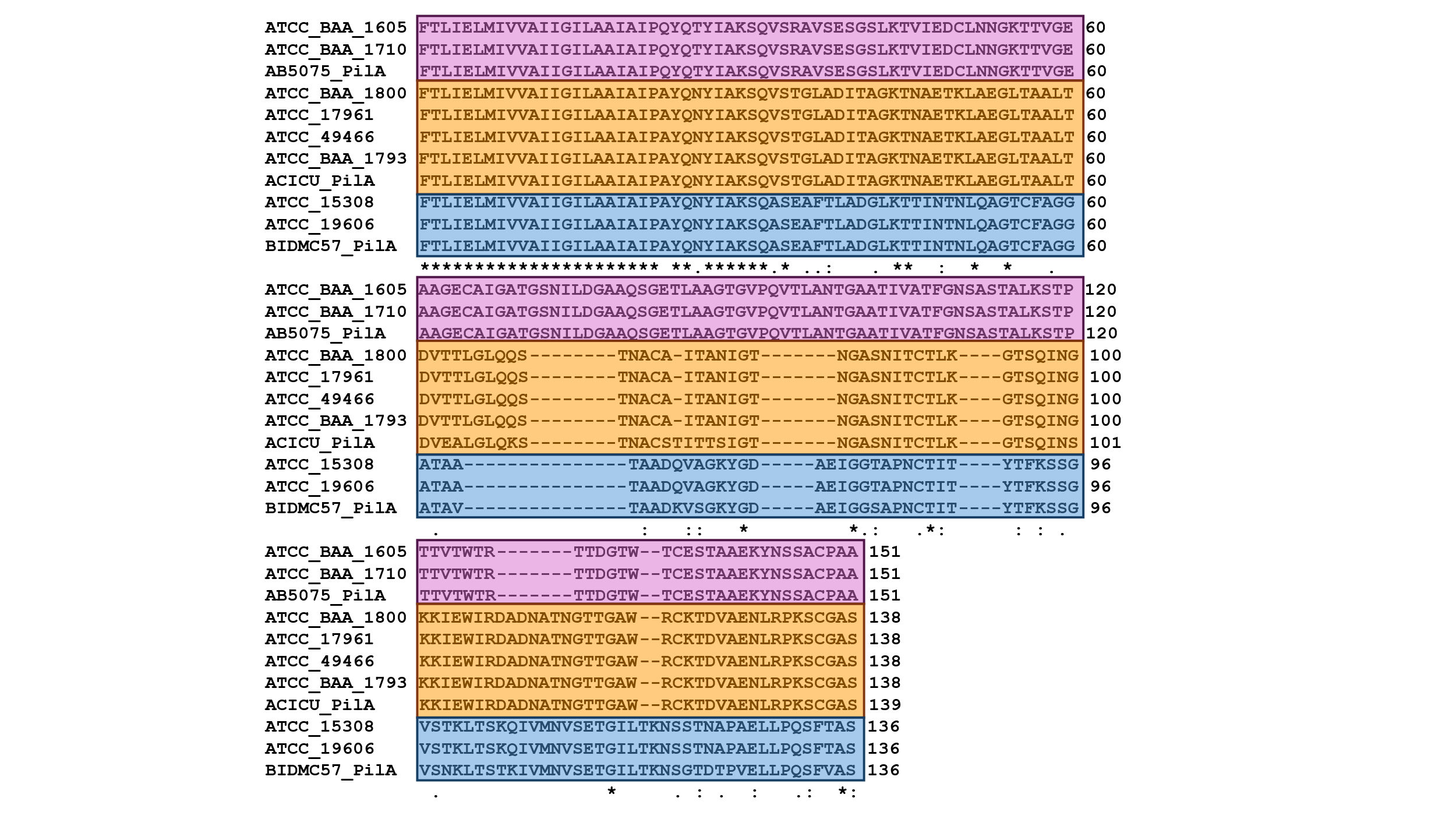


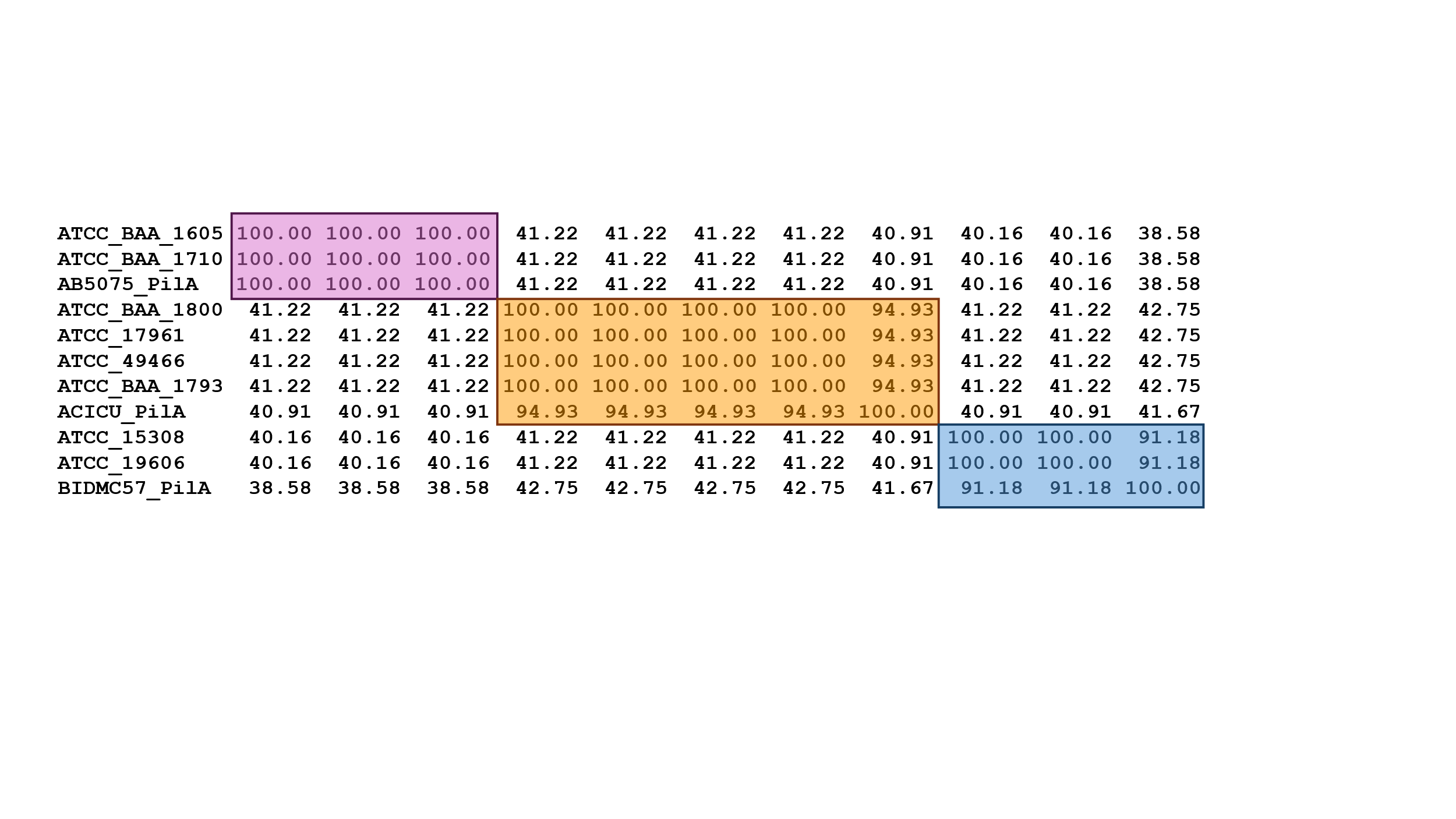


**Supplementary Figure 5:** Alignment of PilA sequences from selected *A. baumannii* strains. The alignment was produced using CLUSTAL Omega (*2*). Sequences are highlighted in color based on similarity to *pilA*^ACICU^ (orange), *pilA*^AB5075^ (pink) and *pilA*^BIDMC57^ (blue).


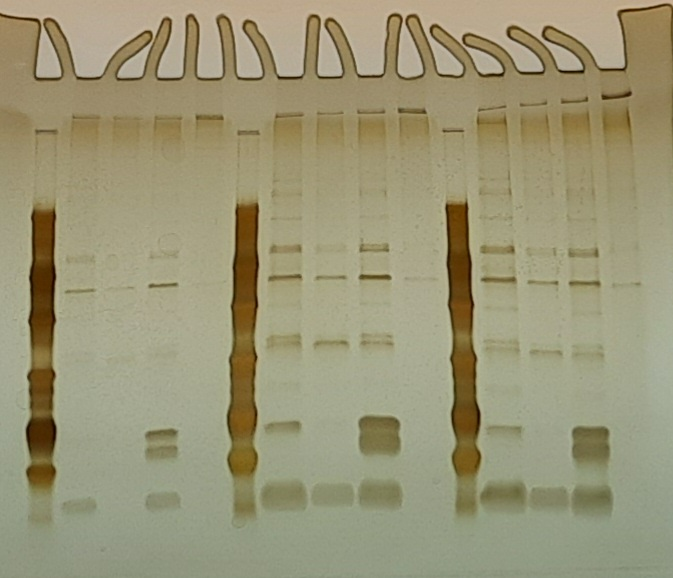


Wild Type

Tn-*pilT*-

Tn-*pilA*-

**Supplementary Figure 6:** Isolation of type IV pili from *A. baumannii* AB5075-UW.. Silver-stained gels are shown for pilus preparations (sheared and precipitated as described in Methods) for *A. baumannii* AB5075-UW wild type and Tn-*pilT*-. The band for pilA (with and without glycosylation) is highlighted with a pink box and the band chosen for the loading control is highlighted with a black box.


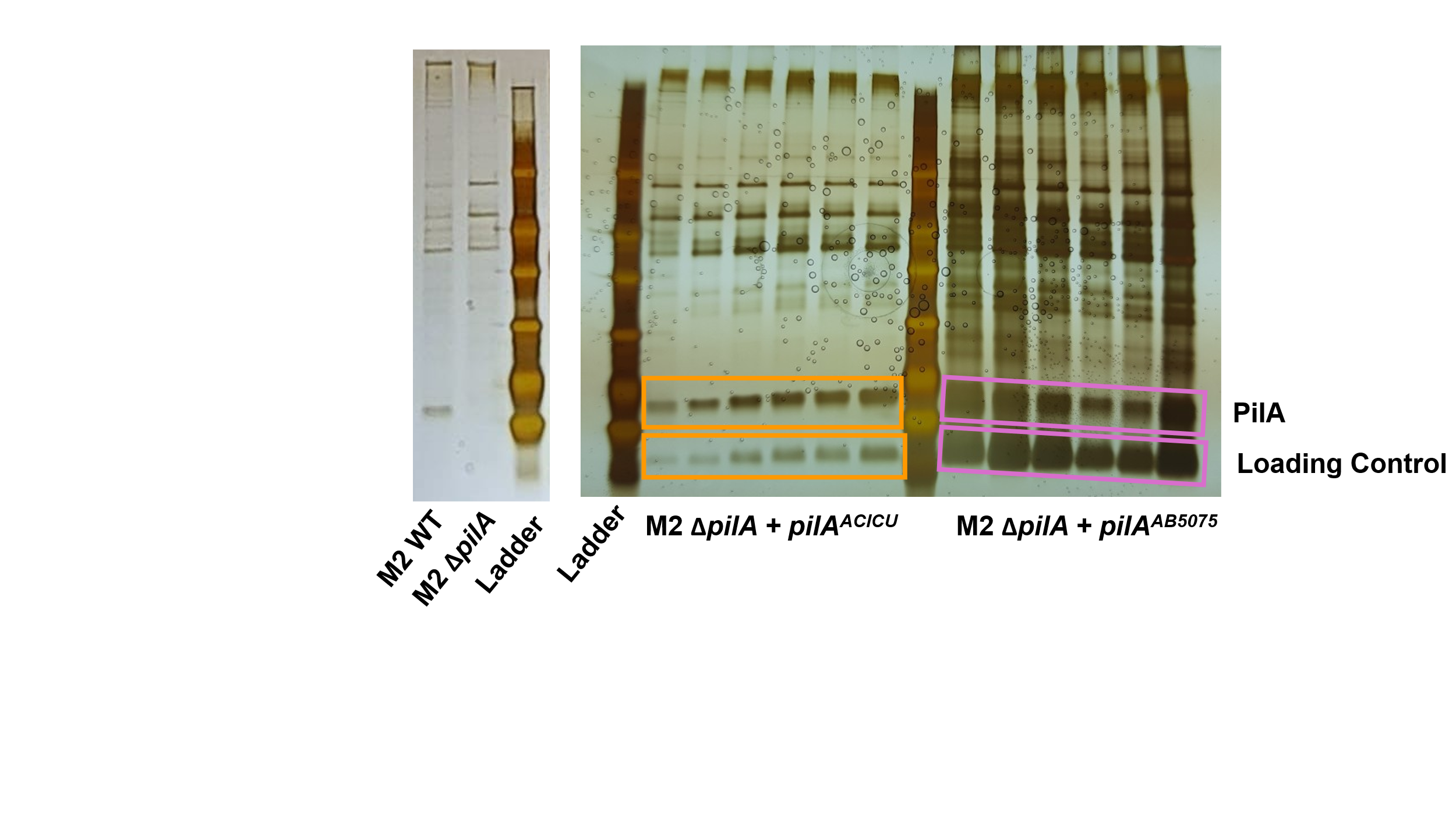


**Supplementary Figure 7:** Isolation of type IV pili from *A. nosocomialis* M2. Silver-stained gels are shown for pilus preparations (sheared and precipitated as described in Methods) for *A. nosocomialis* M2 wt, Δ*pilA* and complements with either *pilA*^ACICU^ or *pilA*^AB5075^. Six biological replicates are shown for each of the complements.

| Construct | Vector | Sequence |
| --- | --- | --- |
| *pilA*^ACICU^ | pUCP20GM | ATGAATGCACAAAAAGGTTTTACATTAATCGAACT  CATGATCGTAGTTGCCATTATTGGTATTTTGGCTG  CGATTGCGATTCCTGCTTATCAAAACTACATTGCT  AAGTCACAAGTAAGTACTGGTTTAGCTGATATTAC  TGCTGGTAAGACAAACGCAGAAACTAAATTAGCAG  AAGGTTTAACTGCGGCATTAACTGATGTAGAAGCT  TTAGGCTTACAAAAATCTACGAATGCTTGTAGTAC  TATTACAACCAGTATCGGAACTAATGGTGCAAGTA  ATATTACTTGTACATTGAAAGGTACATCACAAATT  AATAGTAAAAAAATTGAATGGATCCGTGATGCAGA  TAATGCTACAAATGGTACGACAGGTGCTTGGCGCT  GTAAAACTGATGTAGCTGAAAACTTACGTCCTAAA  TCATGTGGTGCTTCTTAA |
| *pilA*^AB5075^ | pUCP20GM | TTCACTCTGATTGAACTGATGATTGTTGTTGCAAT  TATTGGCATTTTAGCAGCTATTGCTATTCCACAAT  ATCAAACCTATATTGCAAAAAGCCAAGTTTCTCGT  GCTGTTAGTGAAAGCGGTTCTTTAAAAACAGTTAT  TGAAGATTGTCTGAATAATGGCAAAACCACAGTTG  GTGAAGCAGCTGGCGAATGCGCAATTGGTGCTACC  GGCTCAAATATTTTAGATGGTGCAGCTCAAAGTGG  CGAAACTTTAGCAGCTGGTACCGGCGTTCCACAAG  TTACATTAGCAAATACTGGTGCAGCTACCATTGTT  GCTACATTTGGCAATTCAGCAAGTACAGCTTTAAA  AAGCACTCCTACTACCGTTACCTGGACACGTACAA  CTGATGGTACTTGGACCTGTGAATCTACAGCAGCT  GAAAAATATAACTCTTCAGCTTGCCCTGCAGCT |
| *pilA*^ACICU-negative^ | pUCP20GM | TTTACATTAATCGAACTCATGATCGTAGTTGCCAT  TATTGGTATTTTGGCTGCGATTGCGATTCCTGCTT  ATCAAAACTACATTGCTAAGTCACAAGTAAGTACT  GGTTTAGCTGATATTACTGCTGGTAAGACAAACGC  AGAAACTAAATTAGCAGAAGGTTTAACTGCGGCAT  TAACTGATGTAGAAGCTTTAGGCTTACAAAAATCT  ACGAATGCTTGTAGTACTATTACAACCAGTATCGG  AACTAATGGTGCAAGTAATATTACTTGTACATTGA  AAGGTACATCACAAATTAATAGTACAACTATTGAA  TGGATCCGTGATGCAGATAATGCTACAAATGGTAC  GACAGGTGCTTGGACATGTGAAACTGATGTAGCTG  AAAACTTAAACCCTAAATCATGTGGTGCTTCT |
| *pilA*^ACICU-loop swap^ | pUCP20GM | ATGAATGCACAAAAAGGTTTTACATTAATCGAACT  CATGATCGTAGTTGCCATTATTGGTATTTTGGCTG  CGATTGCGATTCCTGCTTATCAAAACTACATTGCT  AAGTCACAAGTAAGTACTGGTTTAGCTGATATTAC  TGCTGGTAAGACAAACGCAGAAACTAAACTGAATA  ATGGCAAAACCACAGTTGGTGAAGCAGCTGGCGAA  TGCGCAATTGGTGCTACCGGCTCAAATGCTTGTAG  TACTATTACAACCAGTATCGGAACTAATGGTGCAA  GTAATATTACTTGTACATTGAAAGGTACATCACAA  ATTAATAGTAAAAAAATTGAATGGATCCGTGATGC  AGATAATGCTACAAATGGTACGACAGGTGCTTGGC  GCTGTAAAACTGATGTAGCTGAAAACTTACGTCCT  AAATCATGTGGTGCTTCTTAA |

**Supplementary Table 1:** Nucleotide sequences for *Acinetobacter baumannii* *pilA* genes *pilA*^ACICU^, *pilA*^AB5075^, *pilA*^ACICU-negative^ and *pilA*^ACICU-loop swap^.

**References:**

1. C. M. Harding *et al.*, Acinetobacter baumannii strain M2 produces type IV pili which play a role in natural transformation and twitching motility but not surface-associated motility. *MBio* **4**, (2013).

2. F. Sievers *et al.*, Fast, scalable generation of high-quality protein multiple sequence alignments using Clustal Omega. *Mol Syst Biol* **7**, 539 (2011).
